# Coenzyme Q and Its Role in the Dietary Therapy against Aging

**DOI:** 10.3390/molecules21030373

**Published:** 2016-03-18

**Authors:** Alfonso Varela-López, Francesca Giampieri, Maurizio Battino, José L. Quiles

**Affiliations:** 1 Department of Physiology, Institute of Nutrition and Food Technology “José Mataix”, Biomedical Research Center (CIBM), University of Granada, Avda. del Conocimiento s.n., Armilla, Granada 18100, Spain; avarelalopez@gmail.com; 2 Dipartimento di Scienze Cliniche Specialistiche ed Odontostomatologiche (DISCO), Facoltà di Medicina, Università Politecnica delle Marche, Ancona 60131, Italy; f.giampieri@univpm.it; 3 Centre for Nutrition & Health, Universidad Europea del Atlantico (UEA), Santander 39011, Spain

**Keywords:** aging, antioxidants, diet, mitochondria, nutrition, oxidative stress, ubiquinone

## Abstract

Coenzyme Q (CoQ) is a naturally occurring molecule located in the hydrophobic domain of the phospholipid bilayer of all biological membranes. Shortly after being discovered, it was recognized as an essential electron transport chain component in mitochondria where it is particularly abundant. Since then, more additional roles in cell physiology have been reported, including antioxidant, signaling, death prevention, and others. It is known that all cells are able to synthesize functionally sufficient amounts of CoQ under normal physiological conditions. However, CoQ is a molecule found in different dietary sources, which can be taken up and incorporated into biological membranes. It is known that mitochondria have a close relationship with the aging process. Additionally, delaying the aging process through diet has aroused the interest of scientists for many years. These observations have stimulated investigation of the anti-aging potential of CoQ and its possible use in dietary therapies to alleviate the effects of aging. In this context, the present review focus on the current knowledge and evidence the roles of CoQ cells, its relationship with aging, and possible implications of dietary CoQ in relation to aging, lifespan or age-related diseases.

## 1. Coenzyme Q: Structure, Localization and Forms

Coenzyme Q (CoQ) is a naturally occurring molecule formed from the conjugation of a benzoquinone ring with a hydrophobic isoprenoid chain of varying chain length, depending on the species [1,2,3]. It is mainly located in the hydrophobic domain of the phospholipid bilayer of the inner membrane system of the mitochondria, but it is also present in all the other biological membranes at significant levels [4,5,6,7,8,9], as well as in plasma lipoproteins [10]. Moreover, CoQ is found in every plant and animal cell [2,11]. Due to its ubiquitous presence in nature and its quinone structure, it is also called ubiquinone [2,12]. Ubiquinone is referred to as “coenzyme” because of its unique ability to participate in chemical reactions but it remains at steady-state levels in the cell [3,13]. There are different ubiquinone molecules that are classified based on the length of their isoprenoid side chain with a subscript indicating the number of carbons in the chain (CoQ_n_) [1,3]. CoQ_9_ (2,3-dimethoxy-5-methyl-6-noneprenyl-1,4-benzoquinone) is the predominant form in rats and mice, whereas in humans and other long-living mammals, the major homologue is CoQ_10_ (2,3-dimethoxy-5-methyl-6-decaprenyl-1,4-benzoquinone) [2,3,14]. The importance of the length of the polyisoprenyl chain is related to the stability of the molecule within the hydrophobic lipid bilayer. In addition, this feature seems to affect other properties, such as mobility, intermolecular interaction with membrane proteins, and autoxidizability [3,15,16].

**Figure 1:**
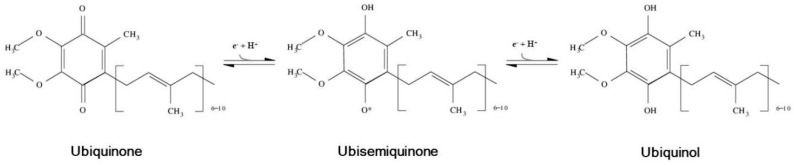
Alternative redox forms of Coenzyme Q.

The benzoquinone ring of CoQ can assume three alternate redox states due to the existence of different possible levels of protonation yielding three alternative CoQ forms (Figure 1): the fully oxidized (CoQ) or ubiquinone, the fully reduced (CoQH_2_) or ubiquinol and the partially reduced (CoQH) or ubisemiquinone [17,18]. Because of its extreme hydrophobicity, it is possible to find natural CoQ in three physical states: dissolved in lipid bilayers, forming micellar aggregates, or bound to proteins. In cells, CoQ is distributed between the two first states [18], whereas the importance of the last one is only experimental [17]. All cells are able to synthesize functionally sufficient amounts of this molecule under normal physiological conditions [19]. However, the content of CoQ as well as the ratios between its forms are different depending on the analyzed species, tissue or even organelle. For instance, murine kidney and heart show higher CoQ levels than brain or liver homogenates [20,21]. Likewise, it has been reported that lysosomes and Golgi membranes contain relatively higher concentrations of CoQ than mitochondrial membranes or microsomes [22,23]. In non-mitochondrial biological membranes, CoQ continuously cycles between reduced and oxidized states thanks to different enzymes with CoQ reductase activity [24]. These enzymes are NAD(P)H dehydrogenases which form part of the plasma membrane redox system, an electron transport where CoQ acts as a mediator accepting electrons from cytosolic NAD(P)H [6]. This system has been related to the maintenance of intracellular redox homeostasis, membrane antioxidant protection, regulation of cell signaling and other functions [6] that will be discussed below.

## 2. Physiological Roles of CoQ

### 2.1. Mitochondrial CoQ Is an Essential Factor for Cell Bioenergetics

**Figure 2:**
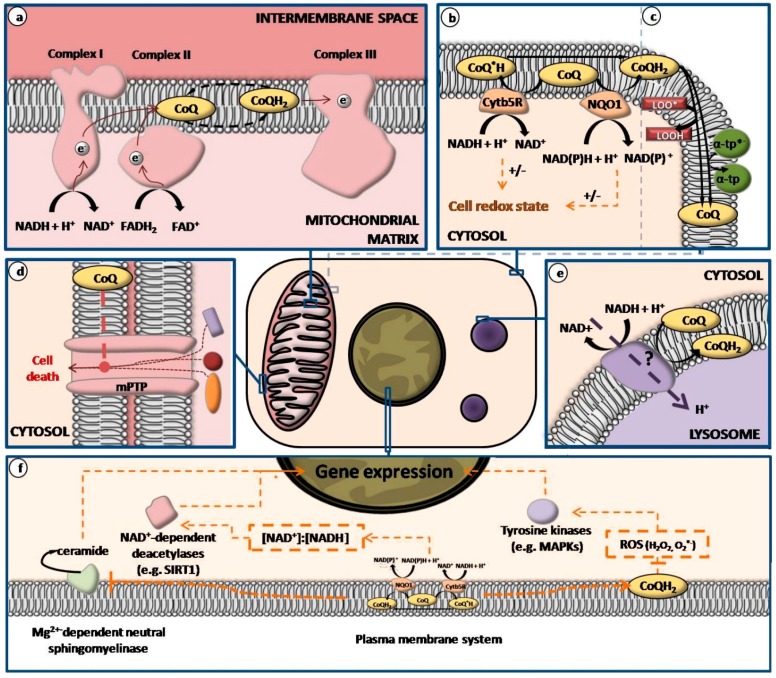
Different activities exerted by coenzyme Q in cells: (**a**) coenzyme Q has a central role in mitochondrial electron transport chain; (**b**) plasma membrane redox system components; (**c**) antioxidant roles of coenzyme Q (this can occurs in all membranes where ubiquinol is present); (**d**) coenzyme Q prevent permeability transition pore opening; (**e**) coenzyme Q participates in lysosomal pH maintenance; (**f**) coenzyme Q stimulatory (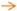) or inhibitory (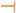) effects on gene expression. Abbreviations: α-tp: α-tocopherol; α-tp*^−^: α-tocopheryl anion radical; CoQ: oxidized coenzyme Q; CoQ·H: partially reduced coenzyme Q; CoQH_2_: fully reduced coenzyme Q; Cytb5R: NADH: cytochrome b5 reductase; e^−^: electron; FAD^+^: reduced flavine adenine dinucelotide; FADH_2_: reduced flavine adenine dinucleotide; H_2_O_2_: hydrogen peroxide; LOO*: lipid hydroperoxyl radical; LOOH: lipid hydroperoxyde; MAPKs: mitogen-activated protein kinases; mPTP: mitochondrial permeability transition pore; NAD^+^: oxidized nicotine adenine dinucleotide; NADH: reduced nicotine adenine dinucleotide; NQO1: NAD(P)H:Ubiquinone oxidase 1; O_2_^**·**−^: superoxide anion; ROS: reactive oxygen species; SIRT1: sirtuin1.

CoQ was isolated and characterized in 1955 and only two years later it was shown to be an essential mitochondrial electron transport chain (mtETC) component [25] (Figure 2). Thus, CoQ is an essential factor for cell bioenergetics [6]. It acts as carrier of electrons from respiratory complexes I and II to complex III [23,26,27,28,29]. Additionally, CoQ also accepts electrons from other donors including dihydroorotate dehydrogenase, and acyl-CoA dehydrogenase. Since it has several electron donors but only transfers them to complex III, it may be considered a crossroad in the mitochondrial electron transport activity. Therefore, CoQ continuously cycles between the reduced and the oxidized forms in mitochondria, but it is also bound to proteins and in a membrane free form. The sum of CoQ in any of these possible states constitutes the mitochondrial CoQ pool [23]. In relation to this, Lopez-LLuch *et al.* [6] have proposed that an equilibrated CoQ pool may perform a better electron flow adaptation than a higher or lower CoQ pool by keeping a better mitochondrial homeostasis control. In addition, it has been reported that protein-bound CoQ participates in and is needed for the maintenance of complex III stability in yeast [30]. According to this finding, CoQ levels in mitochondria would not only affect cell bioenergetics by its participation as coenzyme in mtETC, but also could affect protein complex activity and structure [6]. Moreover, by means of plasma membrane redox system, CoQ is involved in balancing the NAD^+^:NADH ratio in cells, which also regulates cell bioenergetics [31].

### 2.2. CoQ Acts as Lipid-Soluble Antioxidant in All Biological Membranes

One of the main functions of CoQ in biological membranes is as an antioxidant [6] (Figure 2). Furthermore, it constitutes the only lipid-soluble antioxidant endogenously synthesized that has shown to efficiently prevent oxidation of proteins, lipids and DNA [32]. In this role, CoQ can act as antioxidant by itself or as free radical quencher [11,16,33]. It also participates in the regeneration of other antioxidants such as ascorbate [6,34,35] and α-tocopherol [33,36]. In particular, it has been proposed that the antioxidant effect of quinones in mitochondrial membranes is mediated by α-tocopherol recycling [6,37,38]. By itself, reduced CoQ_10_ has also demonstrated to be an important physiological lipid-soluble antioxidant [39]. In contrast to other antioxidants, ubiquinol can inhibit both the initiation and propagation of lipid peroxidation by reacting with perferryl radical and radicals generating ubisemiquinone and a non-radical lipid hydroperoxide [19,25]. Physiological ubisemiquinones having long side chains do not react with oxygen, except in complex III under the very special conditions of the CoQ-cycle [18]. On the other hand, short-chain ubiquinones have a pro-oxidant effect in complex I. Prevention of the lipid peroxidation propagation by the quenching of the initiating perferryl radicals also protects proteins from oxidation. In relation to this, it is also important to point out that ubiquinol effectively regenerates vitamin E from the α-tocopheroxyl radical which additionally contributes to slow the propagation step of lipid peroxidation. Lastly, CoQ has also been shown to prevent DNA base oxidation, which is particularly important in the case of mtDNA [19]. Furthermore, deficiency in some nutrients considered as antioxidants like vitamin E and selenium could be compensated by the induction of the CoQ-dependent antioxidant system [6]. This has been observed in hepatocytes where both, CoQ levels and CoQ reductases, have been shown to be affected by this compensatory mechanism [40]. The latter reinforces the role of CoQ as antioxidant particularly under some physiological situations such as nutritional deficiencies, which is particularly interesting because it is endogenously synthesized. 

Explanations for the exceptionally high efficiency of CoQ as antioxidant include its intramembranous localization, its general and abundant distribution and its effective reduction/reactivation by a number of cellular systems [19,25]. Firstly, its localization is of central significance since ^**·**^OH and O_2_^**·**−^ generated in the membrane would otherwise rapidly react with neighboring lipid and protein molecules that necessitate the availability of effective protective agents close to the site of radical production [19]. Secondly, several effective enzymatic systems catalyze CoQ reduction to achieve its active (reduced) form in eukaryotic cells [32]. In mitochondria, the reduced antioxidant form of CoQ is regenerated by the respiratory chain [19]. Besides the mitochondrial respiratory chain, other enzymes with such function have been found. These include NADH-cytochrome b5 reductase that can reduce CoQ through a one-electron reaction mechanism [40,41] and the soluble enzyme NAD(P)H-quinone oxidoreductase 1 (NQO1) that can reduce quinones by a two-electron reaction [18,42]. This enzyme is induced under oxidative challenge and can maintain the electron flow through the plasma membrane redox system to CoQ when this system is working mainly as antioxidant [43,44]. In addition, a distinct cytosolic NADPH-CoQ reductase different from NQO1 has also been described [38], which seems to be a main factor for non-mitochondrial CoQ reduction [45,46,47]. However, more studies are needed to clarify its role and regulation under oxidative stress [6].

Lastly, it is important to note that ubiquinol is also present in lipoproteins, where it exerts its antioxidant activity. In particular, it has been reported that ubiquinol is the most efficient antioxidant in low-density lipoproteins (LDL) [48] which also contain α-tocopherol [19,48]. As a consequence of LDL protection from oxidation, CoQ would also have anti-atherosclerotic properties. 

### 2.3. CoQ Collaborates in Redox State Regulation by Plasma Membrane Redox System Activity

It has been proposed that plasma membrane redox system activity becomes essential in the maintenance of bioenergetics in cells when activity in mitochondria decreases, as occurs in aging [6,49] (Figure 2). Actually, the plasma membrane redox system is up-regulated in cells lacking functional mitochondria [31,50,51]. In more detail, it has been reported that the amount of CoQ in plasma membrane increases and CoQ-dependent reductases are induced after mtDNA removal that induces the accumulation of cytosolic NADH. This mechanism allows cells to maintain the cytosolic NAD^+^:NADH ratio in response to increase in cytosolic NADH [31,51,52,53] which is necessary for glycolysis to occur correctly. CoQ is also able to act as an oxidant by autoxidation of ubisemiquinone form [11,16,33]. Based on its apparently paradoxical property to potentially act both as a pro-oxidant and an antioxidant, Sohal and Forster [23] suggest that CoQ may also be a modulator of the cellular redox state under physiological and/or pathological conditions affecting the aging process in this way. 

### 2.4. CoQ Participates in Cell Signaling by Plasma Membrane Redox System

The activity of plasma membrane redox system also plays an important role in growth and development of organisms [6,33]. In this sense, CoQ-dependent NADH oxidases of plasma membrane have been shown to be involved in regulation of cell growth and differentiation. In particular, Gómez-Díaz *et al.* [34] found that the activity of CoQ reductases in plasma membrane is modulated during erythrocyte differentiation. Likewise, the vitamin D_3_-induced differentiation of myeloid cells to monocytes is enhanced when this system is activated by ascorbate [53,54]. Similarly, in serum-free cultures, addition of CoQ induces cell growth [55]. In support of the role of plasma membrane redox system in cell signaling, it has been reported that extracellular signaling molecule growth factors, insulin and pituitary extracts activated NADH oxidoreductases of plasma membrane [56]. At least in the case of myeloid cell differentiation, it has been observed that modulation of the differentiation program by plasma membrane redox system activation includes modulation of intracellular second messengers and regulation of the activity of transcription factors [54,57]. Findings from studies on this topic suggest different mechanisms by means of which CoQ might exert these effects on cell signaling, which include the following.

1. Reactive oxygen species (ROS): As previously stated, NAD(P)H dehydrogenases found in plasma membrane, cytochrome b5-reductase and other NAD(P)H dehydrogenases found in plasma membrane, reduce CoQ in a one-electron mechanism yielding semiquinone forms [58]. These CoQ forms have pro-oxidant activity of generating O_2_^**·**−^ or H_2_O_2_ that would act as second messengers on cell signaling mechanisms. Consequently, these would modulate different cell responses affecting cell growth and differentiation processes [59].
2. Tyrosine kinases: It has been suggested that the redox state of CoQ in plasma membrane could control the activity of tyrosine kinases indirectly by generation of H_2_O_2_ and further inactivation of protein phosphatases, or directly by induction of redox-dependent conformational changes [6,60].
3. Voltage-dependent anion channel (VDAC) proteins: As in mitochondria, plasma membrane also contains proteins belonging to the VDAC protein family. One of the components of this family, VDAC1, can function as NADH-ferricyanide reductase, an activity associated to the plasma membrane redox system [61]. Taken into consideration that CoQ is involved in the regulation of VDAC/permeability transition pore in mitochondria [62], López-Lluch *et al.* [6] have suggested a putative relationship of the activity of CoQ in plasma membrane and cell signaling linked to VDAC1 to be considered in future research.
4. NAD+-dependent deacetylases: This group of proteins implicated in genetic expression, such as sirtuins, could be affected and regulated in some manner by the activity of NADH-dependent reductase in plasma membrane [63]. It has been proposed that the variations in activity of CoQ-dependent NADH oxidoreductases in the different biological membranes could also regulate sirtuins because of the effects on redox state [6].
5. Mg^2+^-dependent neutral sphingomyelinase: It has been reported that CoQ-dependent plasma membrane redox system is involved in inhibition of Mg^2+^-dependent neutral sphingomyelinase after oxidative stress [64,65,66]. This is an integral plasma membrane protein involved in the release of ceramide from plasma membrane sphingomyelin and participates in cell signaling, apoptosis, and the modulation of cell responses [6,67].

### 2.5. CoQ Exerts Anti-Inflammatory Effects through Its Antioxidant Activity

CoQ exerts multiple anti-inflammatory effects by influencing the expression of Nuclear factor κB (NF-κB)1-dependent genes [68]. Likewise, H_2_O_2_ has been identified as an activator of the pro-inflammatory trancription factor NF-κB [69]. In view of the antioxidant properties of the reduced form of CoQ_10_ and the effective enzymatic conversion of oxidized CoQ_10_ into its reduced form, CoQ_10_ might mediate its observed anti-inflammatory effects via gene expression (Figure 2). For the study of the expression of genes implicated in inflammatory processes, *in vitro* models based on cells triggered with lipopolysaccharide (LPS) have aroused interest. This is because this bacterial product induces downstream signaling cascades of the transcription factor NF-κB, which in turn leads to the induction of inflammatory genes. In this context, it has been reported that CoQ_10_ down-regulates LPS-inducible genes in the monocytic cell line THP-1, presumably due to its antioxidant impact on gene expression [70]. Thus, as Genova and Lenaz [18] indicated, it is likely that all effects of CoQ at the genetic level may be mediated by its antioxidant effect.

### 2.6. Mitochondrial CoQ Prevents Events Leading to Programmed Cell Death

Different studies have confirmed the protective role of CoQ_10_ against apoptosis by inducing the inhibition of cell death independently from its free radical scavenging properties or antioxidant effects [62,71,72]. Presumably, this occurs by direct inhibition [62] of the opening of the permeability transition pores a high-conductance protein channel located in the internal mitochondrial membrane [73] (Figure 2). Permeability transition pore opening by its inducers has shown to lead to cell death [74]. Opening of the mitochondrial membrane transition pore depolarizes the mitochondrion [18], but also allows the translocation of molecules as large as 1500 Da in size [62] leading to the release of proteins present in mitochondrial intermembrane space into the cytosol. These last constitute different factors that trigger the process of programmed cell death like cytochrome c [18]. Thus, CoQ would counteract other downstream apoptotic events such as ATP depletion, caspase-9 activation, and DNA fragmentation [75,76]. Moreover, the increase of mitochondrial CoQ levels has been related to the lack of induction of permeability transition pore opening in diabetic rats [77].

To elucidate the possible mechanism under this effect, it is important to point out that permeability transition pore shows an ubiquinone binding site where different (natural and artificial) forms of CoQ interact stabilizing the pore in the closed conformation [75,76]. In particular, quinones have also been shown to exert a direct effect on permeability transition pores. These compounds are able to modulate the permeability transition pore through a common binding site rather than through oxidation-reduction reactions. Occupancy of this site can modulate the permeability transition pore open-closed transitions, possibly through secondary changes of the Ca^2+^-binding affinity for the pore [18]. Based on their relation with permeability transition pores, Walter *et al.* [76] distinguish three functional classes of quinone analogs:

- permeability transition pores inhibitors, like CoQ_0_ ,CoQ_2_, and decylubiquinone.
- permeability transition pores inducers, like idebenone 2,3-dimethoxy-5-methyl-6-(10-hydroxydecyl)-1,4-benzoquinone).
- permeability transition pores-inactive quinones, which counteract the effects of both inhibitors and inducers, such as CoQ_1_.

An indirect study [78] suggests that CoQ_10_ may be a permeability transition pore inhibitor. These authors exposed SHSY5Y neuroblastoma cells to neurotoxic β-amyloid peptides and oxygen-glucose deprivation to investigate the neuroprotective effect of CoQ_10_. In these neuronal cells CoQ_10_ increased resistance against β-amyloid peptides-induced cell death that was related to the regulation of permeability transition pore opening. In addition, a decrease of O_2_^**·**−^ production was also noted [78]. Similar studies indicated a protective effect of CoQ_10_ on permeability transition pore opening against amitriptyline toxicity [79]. However, because ROS signaling could be implicated in permeability transition pore opening, it is not yet fully clear whether these CoQ_10_ effects on the transition pore depend on a direct mechanism or are mediated by the antioxidant effect [18].

### 2.7. CoQ Present in Lysosomal Membrane Participates in pH Maintenance

The NADH-dependent CoQ reductase present in lysosomal membrane is implicated in H^+^ translocation from cytosol to lysosomal lumen by means of an ATP-independent mechanism having O_2_ as the final electron acceptor [80] (Figure 2). As expected, this activity contributes to the maintenance of acidic pH into lysosomes. However, the exact role of this enzyme on lysosomal activity is currently under study [6,81].

## 3. Endogenous and Exogenous Sources of CoQ

### 3.1. CoQ Biosynthesis

CoQ endogenous bionsynthesis requires the synthesis of both a benzoquinone ring and an isoprenoid side chain. The precursor of the benzoquinone ring is 4-hydroxybenzoate that is synthesized from tyrosine or, at least theoretically, from phenylalanine [19]. Meanwhile, the isoprenoid side chain is synthesized by a series of reactions starting from acetyl-CoA and ending up with farnesyl pyrophosphate (farnesyl-PP), which comprises the mevalonate pathway [24,82,83]. Consequently, some reactions involved in CoQ bionsynthesis are shared with cholesterol and other lipid compounds [82]. Both the final product of this pathway, farnesyl-pyrophosphate (farnesyl-PP), and the intermediary of this pathway, isopentenyl pyrophosphate (isopentenyl-PP) are utilized for the synthesis of the isoprenoid side-chain of CoQ [19]. Then, the long isoprenoid side-chain of CoQ (which contains 6–10 isoprene units in different species) is synthesized by trans-prenyltransferase, which condenses farnesyl-PP with several molecules of isopentenyl-PP, all in the trans configuration [84]. 

Then, the 4-hydroxybenzoate-polyprenyl transferase, which is encoded by the gene Coq-2 in humans [85], acts by catalyzing the condensation of the isoprenoid side-chain with 4-hydroxybenzoate. After condensation, the benzoquinone ring undergoes a sequence of modifications including *C*-hydroxylations, decarboxylation, *O*-methylation and *C*-methylation to synthesize CoQ. This sequence has been studied mainly in bacteria and yeast. Meanwhile, in mammals, various functions of genes possibly implicated in these modifications have been established through the complementary recognition in yeast [19]. At the moment, six genes encoding different enzymes that catalyze reactions in this sequence have been identified in humans (termed from Coq-3 to Coq-8) [11,12]. However, full details of the synthesis of CoQ in animal tissues have not yet been clarified [19,86].

Endogenous CoQ_10_, synthesized using the radiolabelled precursor ^14^C-para-hydroxybenzoate (pHB), was first detected in mitochondria and later incorporated into mitochondria-associated membranes and endoplasmic reticulum, from where it is transported to other membranes in the cell [87]. This suggests that CoQ biosynthesis mostly occurs in mitochondria but it is redistributed to other organelles. Although this process is largely unknown, as mentioned, variations in the amounts present in different cellular organelles have been observed, with mitochondria, lysosomes and Golgi vesicles showing the highest concentrations [11]. Despite the fact that all cells are able to produce CoQ, its distribution is not uniform among different organs, being present at the highest concentrations in heart, kidney and liver [88]. Likewise, CoQ amounts can also vary among different tissues and structures of the same organ as occurs in bovine brain where CoQ_10_ levels range from 3 pg/g in the white matter to 25 pg/g in the striatum [89]. Moreover, interindividual variations in total CoQ_10_ have been described, as well as a significant difference in total CoQ_10_ between healthy males and females [90]. All these variations would indicate that CoQ_10_ concentrations are tightly distributed around a homeostatic set point in both organs and individuals. In any case, all cells should functionally synthesize sufficient amounts of CoQ under normal physiological conditions. Thus, in contrast to cholesterol, no redistribution via uptake from the circulation is required. As already mentioned, the liver releases a certain amount of CoQ that associates with very low-density lipoproteins (VLDLs), but this pool is not redistributed to other organs [19,91].

Under normal circumstances, it has been assumed that there is a lack of dependence on exogenous sources of CoQ, since all tissues are able to synthesize CoQ. However, situations may arise in which some cell synthetic capacity is insufficient to reach its CoQ_10_ requirements. Metabolically active cells presumably have the highest requirements for CoQ_10_ which explains that CoQ_10_ deficiency susceptibility appears to be greatest in them [3,12]. For this reason, deepening on our understanding about CoQ synthesis regulation, it is interesting to deal with different pathologies. Hydroxybenzoate is generally present in excess, so that the rate of this reaction is determined by the availability of the polyisoprenoid chain [19]. Farnesyl-PP produced by mevalonate pathway is precursor for cholesterol, CoQ, dolichol and isoprenylated proteins [82]. Moreover, the intermediary isopentenyl-PP is utilized for the synthesis of the isoprenoid side-chain of CoQ, but also for the synthesis of dolichol. It has been suggested that synthesis of all end-products of the mevalonate pathway is co-regulated since the initial sequence of reactions leading to such lipids is identical [19]. However, terminal regulation must also occur [19], which would explain greatly varying synthesis rates and different amounts found for these lipids [92]. The terminal points of regulation probably involve the branch-point enzymes that have farnesyl-PP as substrate which are considered to be rate-limiting for the terminal portion of the biosynthetic sequences [93]. These are squalene synthase, trans-prenyltransferase, cis-prenyltransferase and farnesyl- or geranylgeraniol-protein transferases for cholesterol, CoQ, dolichol and isoprenylated proteins. Traditionally, a central role in the regulation of the mevalonate pathway has been attributed to 3-hydroxy-3-metylglutaryl-CoA (HMG-CoA) reductase, but it seems that its primary regulatory role falls to cholesterol biosynthesis [19]. Since squalene synthase Michaellis constant (KM) for farnesyl-PP is high, it is expected that the total amount of farnesyl-PP in the cell exerts its major influence on cholesterol synthesis. Therefore, when this substrate concentration decreases, the enzyme is not saturated and the cholesterol synthesis rate is reduced [94]. In contrast, all of the other branch-point enzymes, *i.e.*, trans- and cis-prenyltransferases, farnesyl- and geranylgeraniol-protein transferases, exhibit low KMs and remain saturated even when the farnesyl-PP pool is smaller.

As in other metabolic pathways, it seems that endogenous compounds also play regulatory roles on CoQ biosynthesis [19]. Epoxidated derivatives of certain all-trans polyisoprenoids, solanesol, tocotrienols, vitamin K_2_ and CoQ up-regulate CoQ synthesis in HepG2 cells by themselves. Additionally, it has been reported that several of these compounds inhibit the biosynthesis of cholesterol in the same cultures [95]. However, none of the epoxidated poly-cis polyisoprenoids, which occur naturally without an epoxy group in large numbers, exhibit any effect on products of the mevalonate pathway in this same system [19]. Tocotrienols exert the most profound effects on this biosynthetic pathway. It has been observed that dolichol synthesis is also up-regulated but the importance of this phenomenon has not been clarified since the function for non-phosphorylated form in animals has not been established despite its abundance. In general, improved CoQ biosynthesis is achieved by regulating entire biosynthetic machinery, while decreasing cholesterol synthesis specifically involves the inhibition of oxidosqualene cyclase. Therefore, it has been suggested that small amounts of mono- and diepoxides polyisoprenoids, as those having many organisms, could be biological regulators of the mevalonate pathway [19]. At translational level, some molecules have been shown to stimulate CoQ_10_ synthesis by Coq-7 expression regulation [96,97]. In particular, it has been found that CoQ_10_ biosynthesis is dependent on NF-κB, which binds specifically to two binding sites present in the 5’-flanking region of the Coq-7 gene, inducing both the Coq-7 expression and CoQ_10_ biosynthesis [98].

### 3.2. Dietary CoQ Has Shown to Increase CoQ Levels in Different Body Compartments

CoQ is a molecule naturally found in different dietary sources, which can be taken up from intestinal lumen in a similar way to other lipids [83]. According to results from different studies in rats [96,97,98,99,100,101,102,103], it has been historically considered that approximately 6% of orally administered CoQ permeates the gastrointestinal tract into the blood and is transferred to liver and spleen. Consequently, uptake in the whole body ranged between 2% and 3% of the total dose. Moreover, CoQ_10_ has been found in plasma, largely in the reduced form [100]. *In vitro* studies with CaCo-2 models of absorption have suggested that CoQ_10_ is reduced to ubiquinol either during or following absorption [104]. In turn, CoQ uptake by other tissues such as heart, kidney, brain and skeletal muscle was considered low or completely absent [100,102,103], unless endogenous levels fall below a critical threshold. 

In humans, different supplementation studies have been carried out [105,106,107,108,109,110,111,112,113,114] varying in duration (20 days, 3 or even 16 months) and dosages (100–2400 mg/day), as well as in experimental group’s characteristics. Despite the differences, all showed an increase of CoQ_10_ levels in blood. Further, such increase seems to be a dose-dependent one according to a study where the effects of supplementation with 90, 150, and 300 mg/day of the reduced form of CoQ_10_ were compared [108]. In elderly women it was reported that the change was inversely associated with the baseline concentration [107], which suggests that CoQ uptake is also affected by levels present in the body. However, when CoQ_10_ concentration was assessed in other samples (homogenized muscle and mitochondria), no increase was noted [105]. Regarding how duration of dietary treatment affects CoQ body levels, it was observed that plasma ubiquinol concentration nearly reached steady-state by two weeks after the start of treatment, and plasma ubiquinol levels returned to those before administration six months after completion of treatment [108]. In another study [109], maximum plasma CoQ_10_ concentration was reached in two weeks of supplementation with all-trans form of CoQ_10_ and then decreased to basal level after withdrawal, but CoQ_10_ levels increased by a third compared to the study by Hosoe *et al.* using ubiquinol [108].

Results from more recent studies in mice and rats [20,21,110,111,115,116,117,118,119,120,121,122] firmly refute this long-held notion that CoQ content of tissues other than plasma, liver or spleen cannot be significantly augmented by dietary administration of CoQ_10_. It was first shown by Matthews *et al.* [110] that CoQ_10_ intake in diets for two months by 12- or 24-months-old rats increased CoQ content in brain mitochondria. Subsequently, a series of studies conducted by Sohal *et al.* demonstrated that CoQ_10_ administration (from 0.7 to 370 mg/Kg per day) via food to mice [20,21,116,120,121] and rats [113,115,116,117,118,119] caused an increase in amounts of CoQ_10_ in plasma and in tissue homogenates and mitochondria of brain, heart, kidney, skeletal muscle and liver. In all tissues, the amount of CoQ augmentation was greater in mitochondria than in the homogenate, suggesting its preferential sequestration in mitochondria [6]. In brain, the increase was of a lesser magnitude and occurred primarily in mitochondria and not in the homogenate. With this background, it seems more clear that different tissues tend to vary in their capacity for CoQ accretion, with liver and skeletal muscle exhibiting the highest elevations, and brain showing the lowest [23]. The mentioned studies in animals were performed with relatively high daily dietary amounts of CoQ suggesting that higher plasma CoQ_10_ concentrations are necessary to facilitate uptake by peripheral tissues [83,116], which could explains differences with earlier studies. However, it has also been observed that feeding rats with very low-dosages of CoQ_10_ from weaning leads to increase of mitochondrial CoQ levels of heart and liver in 12- and 24-months-old rats. Moreover, it has been observed that this difference observed between rats increases as the animals aged [118,123]. Thus, age at beginning and duration of the treatment must be taken into account too. Importantly, dietary CoQ_10_ also leads to an increase of the rat specific form CoQ_9_ in all tissues where it was measured [20,21,118].

Notwithstanding, as Sohal and Forster [23] pointed out, the uptake of CoQ and other lipid substances is a complex process dependent upon a considerable number of different factors. In that sense, it has been reported that intestinal absorption is threefold faster if CoQ_10_ is administrated with food intake in rats [124]. In mice, the increase of plasma levels per 100 mg values was remarkably higher with the reduced form of CoQ_10_ when compared to the results obtained with the oxidized form CoQ_10_ [22]. Additionally, other aspects of CoQ form also seem to be important. Villalba *et al.* [83] reviewed the efficacy of a variety of commercial formulations that have been developed to solubilize CoQ_10_ and promote its better absorption *in vivo*, and its use in the therapy of pathologies associated with low CoQ levels with emphasis in the results of the clinical trials. They concluded that the relative bioavailability of CoQ_10_ is dependent on the type and amounts of oil in the formulations as well as on its delivery system, the order of decreasing bioavailability being: nanoparticulated, solubilized, oil-emulsioned and powder. Some additional dietary factors have been shown to influence CoQ contents in the body and they could also influence the magnitude of dietary CoQ effect. In that sense, it has been reported that monounsarturated fatty acids (MUFA)-rich dietary fats increased CoQ mitochondrial contents, whereas these decreased in diets rich in polyunsarturated fatty acids (PUFA) [125]. In the same sense, it has been observed that a diet rich in saturated fat for 50 days decreased CoQ levels in plasma and liver mitochondria of rabbits, which was restored by oral administration of soluble CoQ_10_ (25 mg/kg per day) over 30 days after such period [126].

Regarding CoQ supplements safety, different assessments in human and animals reviewed by Hidaka *et al.* [127] concluded that the endogenous biosynthesis of CoQ_10_ is not influenced by exogenous inputs. Moreover, it does not accumulate into plasma or tissues when supplementation ends. In rats, a 52-week chronic toxicity study has indicated that the acceptable daily intake is 12 mg/kg per day, calculated from the no-observed-adverse-effect level of 1200 mg/kg per day. In humans, clinical trials suggest an observed safety level for CoQ_10_ was 1200 mg/day per person. In addition, doses up to 3000 mg/day of CoQ_10_ did not cause serious adverse effects in humans. However, several moderate adverse effects as nausea and other adverse gastrointestinal effects have been reported, although these effects were not causally related to the active ingredient because there was no dose-response relationship [127]. At very high amounts (2.6 mg/g), prolonged intake of CoQ_10_ has shown to exacerbated cognitive and sensory impairments in old mice. In turn, intake at lower amounts had no discernable negative impact on these functions [128].

## 4. Evidence for CoQ as Anti-Aging Compound 

### 4.1. CoQ Levels Are Affected by Aging

It has been proposed that along the entire life of organisms, the synthesis of CoQ changes and is significantly reduced from the initial phases of aging [10,129]. This statement seems to contradict findings from *C. elegans* where older individuals showed increases in CoQ content [130]. However, measures of CoQ amount in mammals, mostly rodents, have differed. In mice, age-related decreases of CoQ levels have been observed in liver homogenates [130] as well as in mitochondrial CoQ levels of skeletal muscle, but not of kidney, brain or heart [120]. Like in mice, liver CoQ levels also decrease with age in pigs (*Sus scrofa domestica*) [130]. However, mitochondria of liver, heart and kidney from 19-month-old *ad libitum* fed rats showed lower CoQ_9_ than those from 4-month-old animals, although this CoQ depletion was not detectable in tissue homogenates [131]. Authors attributed this fact to the preferential sequestration of CoQ in the mitochondrial fraction. Similarly, no changes in the levels of CoQ in rat brain after an increase during the first months of life have been reported [132], but in this organ no changes were observed when only mitochondria were evaluated [133]. On the other hand, an age-associated decrease of CoQ concentration was detected in pancreas and adrenal gland, brain, heart and lung after an early increase like in brain reaching highest values at 30 days of age [134]. In an old study, CoQ increased between 2 and 18 months and decreased significantly at 25 months in the heart and kidney, and the gastrocnemius, oblique and deep aspect (red) of vastus lateralis muscles. On the other hand, CoQ concentration of rat liver increased over the life span, while it remained relatively constant in brain, lung, and the superficial aspect (white) of the vastus lateralis muscle [135]. These results suggest that decrease could appear relatively later in life explaining why some studies do not find clear age-associated changes.

Results of the various studies described do not support the existence of a common trend for all living organisms, although age-associated changes in CoQ content are most evident in mitochondria as was suggested by Sohal and Forster [23]. In mammals, CoQ levels tend to decrease with aging, but this depends on tissues and organisms and probably many other factors. This could explain, at least in part, the differences found in aging processes among different organisms, and also why some tissues are more susceptible to aging or aging-related diseases. Studies evaluating the association between age and CoQ concentration in humans have also supplied contradictory results. In elderly women, no significant correlations were found between CoQ_10_ plasma levels and age [107]. In human pancreas and adrenal gland, CoQ_10_ levels were highest at one year of age, and then they decrease, whereas in the brain, heart and lung, the corresponding peak value was at 20 years of age and was followed by a continuous decrease upon further aging [134]. Age-associated decrease in brain has been confirmed by later studies [136,137]. In fact, it has been suggested that the mevalonate pathway is affected in the aged brain [137].

### 4.2. Several Age-Related Pathologies Are Associated with Low Levels of CoQ

Diseases such as cardiovascular diseases, neuropathies, inflammation, metabolic syndrome, arthritis, carcinogenesis, diabetes or hypercholesterolemia worsen during aging and are considered as major age-related diseases [6]. Different studies have reported a beneficial effect of CoQ on them [138,139,140,141,142], almost all in animal models. This fact has been taken into account to suggest that CoQ becomes an essential factor in the maintenance of the normal activity of cells in such conditions [6]. However, it is important to note that some interventions in humans in relation to Parkinson disease [112] and Amyotrophic Lateral Sclerosis [113] have not offered clear benefits for CoQ on disease progression.

These and other neurodegenerative diseases have been widely studied in this respect. Case-control studies have reported lower CoQ_10_ levels in serum of Lewy’s body disease patients [143] and plasma of subjects with amyotrophic lateral sclerosis [144] compared with healthy controls. Likewise, lower levels of this molecule have been found in cerebrospinal fluid of Alzheimer´s disease patients [145]. In addition, a negative correlation has been reported between CoQ levels and the duration of illness in Amyotrophic Lateral Sclerosis [145], Alzheimer’s [146] and Parkinson’s disease case studies [147]. These observations, together with the decrease of mitochondrial activity and the increase of free radical levels found in neuroneurodegenerative diseases [147,148,149,150], highlight the importance that CoQ has in these processes as suggested by Lopez-Lluch *et al.* [6]. Actually, it has been reported that NQO1 expression increases during the initial steps of Alzheimer’s disease, indicating a higher lipid peroxidation coupled to a higher necessity for CoQ-dependent antioxidant activity [6,151].

Some authors have also focused their studies on a possible relationship between CoQ amount and some metabolic syndrome components as risk factors of cardiovascular disease. An important component associated to aging is hypercholesterolemia since it affects a significant part of the aged population. In elderly women, no differences were seen between hyperlipidemic and normolipidemic subjects in relation to serum CoQ_10_ levels. Similarly, no significant correlations were found with body mass index, another risk factor for cardiovascular disease [108]. However, as indicated above, CoQ is the main antioxidant in LDL [48]. In relation to this, it has been shown that total CoQ_10_: total cholesterol ratio was reduced in diabetes mellitus patients, another component of metabolic syndrome, compared with subjects with normal glucose tolerance and impaired fasting glucose [152]. 

The development of certain types of cancer also could be related with low CoQ levels. Epidemiological studies in humans have shown negative associations between CoQ levels in blood and breast cancer [153,154], myeloma [153], melanoma [155], Graves’ disease or follicular and papillary thyroid carcinomas [156]. Some studies have also suggested that tumoral cells have lower CoQ levels than normal cells. In women presenting carcinomas and non-malignant-breast cancer, CoQ_10_ concentrations in breast tumor tissues significantly decreased when compared to the surrounding normal tissues [157]. Similarly, melanoma cell lines also presented low concentrations of CoQ_10_ [155]. In relation to these groups of diseases, it has been found that CoQ treatment decreases cell growth of a prostate cancer line (PC3 line) but not of non cancer cells (PNT2) and was associated with a high production of ROS [158], suggesting an interesting use for this molecule in cancer therapy.

### 4.3. Aging, Development and Lifespan Are Associated to Changes in CoQ Bionsynthesis

In mammals, there are at least 10 different proteins participating in the biosynthesis of CoQ that are encoded by Coq genes. These have both catalytic and regulatory activities. The first group is encoded by the genes Coq-1, Coq-2, Coq-3, and Coq-7, whereas it has been suggested that Coq-4, Coq-8 and Coq-9 gene products have regulatory functions [6,84]. Different studies using animal model mutants for these genes have been carried out to elucidate the role of endogenous CoQ synthesis on aging. *C. elegans* models have been extensively used in this way. clk-1 (mammals Coq-7 orthologue gene) mutant nematodes that produce a very low amount of CoQ [159] showed an increased lifespan compared to wild-type animals [160,161]. However, analysis of mutants in Coq genes, other than clk-1, provided different results. In most of cases, Coq genes knockout showed deleterious defects leading to early developmental arrest [162,163,164]. This has been reported for Coq-1, Coq-3, Coq-3 and Coq-8. Extension of longevity by silencing Coq genes [165] occurs upon moderate low levels of global CoQ content (up to 50%) but not in the case of more severe CoQ depletions as observed in Coq-8 [162] and Coq-3 [164] mutants. To explain this difference it has been proposed that moderated CoQ depletion is associated to lower ROS production extending lifespan [165], whereas a higher CoQ depletion would lead to developmental and reproductive inefficiency [162,164].

According to findings from the studies with *C. elegans*, it has also been suggested that Coq-7 and CoQ synthesis is also related to aging in humans [166] and other mammals. However, experimental evidence suggests that important differences could exist between mammals and *C. elegans* in this sense. In contrast to observations in nematodes, murine Coq-7 knockout embryos arrest development at midgestation [167]. Similarly, it has been reported that some deletions in Coq-7 affect mitochondrial integrity and neurogenesis [168], which is comparable to some effects of CoQ deficiency found in humans [169,170]. However, heterozygous mice for this gene carrying a single functional copy show a notable increase in lifespan [171].

Mutations in genes implicated in CoQ biosynthesis have been identified as a cause of different pathologies associated with CoQ deficiencies in humans, most of which appear early in life [172,173,174,175,176]. The most severe human CoQ_10_ deficiencies are due to autosomal recessive mutations and can be classified as primary deficiencies when mutations affect CoQ_10_ biosynthetic genes, or secondary if the cause is related to other genetic defects [177]. Hereditary CoQ_10_ deficiencies caused by these mutations usually lead to cardiomyopathies and degenerative muscle and neuronal diseases [169,173,178,179,180]. The major phenotypes provoked by CoQ_10_ deficiencies are encephalomyopathy, severe infantile multisystemic disease, cerebellar ataxia, Leigh syndrome with growth retardation, ataxia and deafness, and isolated myopathy [181].

## 5. Studies on Dietary Therapies with CoQ on Aging

According to previous observations, dietary supplementation with CoQ_10_ could constitute an anti-aging strategy. In humans, there is evidence, mainly indirect, that exogenous orally administered CoQ_10_ may be incorporated into mitochondria, at least in conditions of partial CoQ tissue deficiency, where it may enhance electron transfer and ATP synthesis with improvement of pathological situations such as cardiac failure [182,183], Parkinson’s disease [115,184,185,186], Alzheimer’s disease [187,188,189] and Friedreich’s ataxia [190]. 

Results from animal studies are not clear about the dietary CoQ effects on longevity. In *C. elegans*, it has been reported that dietary CoQ prolonged lifespan [162], but this was also noted with a CoQ-deficient diet [191]. One possible explanation for poor diet effect could be in the adaptability of these nematodes to stressful conditions. *C. elegans* life span is extended by the intake of antimycin A, an mtETC inhibitor, whereas this molecule is toxic to most other aerobic species [192]. According to this observation, CoQ deficiency might induce a hypometabolic or a dauer-like state, which would facilitate survival under adverse conditions. However, a study focused on features of bacteria used to feed worms has evidenced that this phenomenon may be more complex. In this referred study, a diet based on respiratory incompetent *E. coli*, regardless if they were CoQ-less or CoQ-replete, produced a robust life extension in wild-type *C. elegans* [193]. An explanation for these observations was that the fermentation-based metabolism of the *E. coli* diet is an important parameter of *C. elegans* longevity [193]. 

As in invertebrates, simple dietary CoQ supplementation has shown no direct concluding results on lifespan extension in rodents. Information from this model indicates that CoQ_10_ supplementation with daily dosages ranged from 10 to 370 mg/Kg has no effect on longevity [117,119,129,194]. Despite the absence of evidence supporting that dietary CoQ can increase lifespan in animals, some interventions in the same way have been found to retard certain aging detrimental aspects in different animal models for aging or age-related diseases. In SAMP mice, a mouse model for accelerated senescence and severe senile amyloidosis, life-long supplementation with CoQH_2_ substantially decreased the senescence grading scores at different ages, although it did not alter some age-associated features of the model like the senile amyloid deposition rate. Again, this intervention did not have an effect on the lifespan [195]. In older mice with clear cognitive and psychomotor impairments, short-time (15 days) CoQ-supplementation improved spatial learning [196]. A cardinal event of diabetes like diabetic neuropathy has also been reported to be positively modified upon CoQ administration in diabetic rats [140]. In the hypercholestolemic ApoE knockout mouse, dietary CoQ had an anti-atherogenic effect preventing the accumulation of lipid peroxides in aorta [139]. In turn, in most of cases beneficial effects of CoQ over mitochondrial function and oxidative stress have been demonstrated [139,143,196,197]. However, long-term CoQ_10_ intake in healthy mice fed a standard diet failed to modulate mitochondrial respiratory capacity in liver or levels of oxidative stress in liver, kidney, skeletal muscle or brain [23,119]. According to different findings, López-LLuch *et al.* [6] suggested that supplementation with CoQ is not needed when the organism is young and healthy because cell membranes seem to be nearly saturated at the functional level. However, this supplementation becomes necessary when the organism shows deficiency, as in aging. This could explain why CoQ effects are clearer in animal models of disease than on lifespan of healthy animals if they are related to low CoQ levels.

On the other hand, the combination of dietary treatments using CoQ supplements in certain nutritional conditions associated with higher oxidative stress levels and age-related detrimental effects offers interesting expectations. From this standpoint, studies comparing CoQ effects between isocaloric diets with different lipid profile are particularly interesting. The effects of long-term supplementation with daily CoQ_10_ at 0.7 mg/kg on rats fed on MUFA-rich diets have been compared with those found in n-6 PUFA-rich diets [118,123,198,199,200,201]. One of the most interesting findings from such studies was that dietary CoQ_10_ produced significant increases of mean and maximum lifespan in rats fed a diet rich in n-6 PUFA [123,199,200]. At the histopathological level, when sunflower oil was the main fat in the diet, CoQ supplementation seemed to improve endocrine pancreas structure and in particular β-cell mass resembling positive effects of virgin olive oil [201]. Similar effects were noted in rat alveolar bone loss associated to aging [202]. Dietary CoQ treatments have also been shown to be effective in counteracting many of the high-fat diet consequences in animals [129,203,204,205,206,207,208]. In other mouse models, post-weaning dietary supplementation with CoQ_10_ rescued many of the detrimental effects of nutritional programming on cardiac aging by low birth-weight and catch-up growth [197].

The biochemical basis of potential beneficial effects of CoQ on lifespan or other aging detrimental effects may include enhancement of the cellular antioxidant protection systems in cell membranes, where CoQ sustains lipids in its reduced redox state preventing lipid peroxidation, particularly the unstable PUFA [6,209]. In previous studies in rats, diets containing CoQ were associated with lower lipid peroxidation markers [118,199], as well as with lower oxidative damage of other macromolecules such as DNA or proteins. A higher antioxidant capacity [199,200] compared to that found in animals maintained on the same diet without additional CoQ_10_ has been reported [118,122,198,202]. In addition, a lower impairment in mitochondrial function was also observed in CoQ-fed animals [118]. All these findings would indicate that dietary CoQ_10_ avoids, at least in part, oxidative stress linked to aging under certain conditions. Furthermore, it has been shown that life-long dietary supplementation with CoQ_10_ attenuated a variety of changes in enzymatic activities associated with aging in rats [209,210]. These include increases in the hepatic activities of Mg^2+^-dependent sphingomielinase [209] and of cytosolic and membrane-bound NQO1 activities [210], as well as decreases in cytosolic glutathione-*S*-transferase and microsomal Se-independent glutathione peroxidase in liver plasma membrane [209]. Proteomic analysis in rats under similar conditions has shown that serum albumin, which decreases with age in the rat, was significantly increased by CoQ_10_ supplementation. Additionally, it induced significant modifications of several proteins in plasma. These modifications support the beneficial role of dietary CoQ_10_ decreasing both oxidative stress and cardiovascular risk, and modulating inflammation and osteogenesis during aging [211].

In humans, some studies have suggested similar effects for dietary CoQ in relation to oxidative stress, at least in combination with certain dietary patterns. Short-term (4 weeks) dietary CoQ effects on aging have been tested in combination with the Mediterranean diet. In this regard, elderly subjects ingested a Western diet rich in saturated fatty acids (SFA), a Mediterranean diet (rich in MUFA), and a Mediterranean diet supplemented with CoQ following a cross-over design [212,213,214,215,216]. CoQ_10_ addition to MUFA-rich diet reduced some postprandial oxidative stress marker levels when subjects took a breakfast with a lipid profile similar to their experimental diets [212], which correlated with a lower expression of antioxidant enzyme components [212,215]. Moreover, dietary CoQ has also been shown to improve DNA repair systems [213,214] and modulate inflammatory signaling cascade as well as to reduce endoplasmic reticulum stress [214]. 

All these results suggest that although CoQ supplementation does not directly extend lifespan, it may help to prevent life span shortening due to oxidative insults [6] as it has been suggested by its effect in all aspect related to mitochondrial function, oxidative stress and antioxidant defenses both in animals [139,196,197] and humans [114,143]. However, despite animal studies have shown certain beneficial effects on the health of different disease models, there are clinical trials reported no significant effects on the progression of some nervous central system disease [113,114]. As has been stated, brain CoQ uptake is very low compared to other organs [23] and animal studies reported beneficial effects on this organ [195,196] have used very high daily dosages in relation to body weight compared with those used in clinical trials [113,114]. Future trials in humans focused on diseases affecting tissues and organs that have shown low CoQ uptake capacity should be more careful at this respect and to use higher CoQ dosages and/or chemical formulation with higher bioavailability such as those nanoparticulated or solubilized [83]. In the same sense, possible differences in bioavailability and efficacy between ubiquinol and ubiquinone should also be taken into account. On the other hand, CoQ effectivity for different disease treatment could depend on many other conditions not considered in clinical trials: among others, possible differences in etiology and pathophysiology among animal models and human diseases.

Due to differences in dosage, duration, chemical formulation and subject age at the beginning of the treatment, it is very difficult to establish “ideal conditions” for CoQ use as “anti-aging therapy”. Notwithstanding, according some to animal studies [118,122,198,202], life-long dietary interventions could result useful to prevent negative consequences of different health insults throughout life, particularly in relation to lifestyle. However, ubiquinone dosages used (0.7 mg/kg) already seem very high to take them. In this sense, supplementing with some foods, particularly those that are more “pro-oxidant”, could result useful.

## Abbreviations

The following abbreviations are used in this manuscript:

CoQ	Coenzyme Q
CoQH	partially reduced coenzyme Q
CoQH_2_	fully reduced coenzyme Q
Cytb5R	NADH: cytochrome b5 reductase
FAD^+^	reduced flavine adenine dinucleotide
FADH_2_	reduced flavine adenine dinucleotide
farnesyl-PP	farnesyl-pyrophosphate(isopentenyl-PP)
HMG-CoA	3-hydroxy-3-metylglutaryl-CoA
LDL	low-density lipoprotein
LOO	lipid hydroperoxyl radical
LOOH	lipid hydroperoxyde
LPS	lipopolysaccharide
MAPKs	mitogen-activated protein kinases
mPTP	mitochondrial permeability transition pore
mtDNA	mitochondrial DNA
mtETC	mitochondrial electron transport chain
MUFA	monounsarturated fatty acid
NQO1	NAD(P)H-quinone oxidoreductase 1
pHB	para-hydroxybenzoate
PUFA	polyunsarturated fatty acids
ROS	Reactive oxygen species
SFA	saturated fatty acids
SIRT1	sirtuin1
VDAC	Voltage-dependent anion channelNuclear factor κB (NF-κB)
VLDL	very low-density lipoprotein
α-tp	α-tocopherol
α-tp^***^−^**^	α-tocopheryl anion radical
